# An infant brucellosis meningitis caused by *Brucella* strain

**DOI:** 10.1016/j.bsheal.2025.03.002

**Published:** 2025-03-22

**Authors:** Guowu Shen, Xiaohua Zhao, Jie Chen, Xuehui Zhang, Xin Wang, Zhiguo Liu, Zhenjun Li, Canjun Zheng

**Affiliations:** a Qinghai Provincial Women and Children’s Hospital, Xining 810007, China; b Comprehensive Technology Service Center of Quanzhou Customs, Quanzhou 362000, China; c National Key Laboratory of Intelligent Tracking and Forecasting for Infectious Diseases, National Institution for Communicable Disease Control and Prevention, Chinese Center for Disease Control and Prevention, Beijing 102206, China; d Chinese Center for Disease Control and Prevention, Beijing 102206, China

**Keywords:** Infant brucellosis, Meningitis, Standard tube agglutination test (SAT), Rose Bengal plate test (RBPT), *Brucella* strain, Diagnosis

## Abstract

Brucellosis poses a significant health threat to the population, particularly to vulnerable populations, including infants. In this investigation, we retrospectively analyzed the infection source and potential transmission route in a three-month-old infant with febrile seizure. Bacteriology methods, epidemiological survey, Rose Bengal plate test (RBPT), and standard tube agglutination test (SAT) were used to diagnose the disease, and matrix-assisted laser desorption/ionization time-of-flight mass spectrometry (MALDI-TOF-MS) was applied to identify the strain. The study revealed that the infant’s parents had been diagnosed with brucellosis due to occupational exposure to infected sheep. The *Brucella* strain was isolated and identified from the infant’s blood sample, confirming brucellosis meningitis. Post-treatment serum analysis showed RBPT positivity and SAT titer of 1:200 (+ +). The infant had no direct contact with livestock, with breast milk as the only dietary source; however, the detailed transmission route remained undetermined. Maternal-fetal transmission or contamination through breastfeeding, parental hand contact, clothing exposure, or other passive contamination modes may be potential transmission routes. Notably, the parents had a history of brucellosis and given that the infant ​presented with a fever of unknown origin, screening for brucellosis should have been prioritized. Following diagnosis, the infant was treated with ceftriaxone sodium (2.0 g/day) and rifampicin (0.5 g/day) for four weeks, ultimately achieving full clinical recovery. This case highlights the importance of brucellosis screening in infants presenting with unexplained fever, especially in families whose members have previously been diagnosed with brucellosis in endemic regions.

## Highlights

• The *Brucella* strain was isolated and confirmed as brucellosis meningitis.
• While the exact transmission route remained unclear, potential vertical transmission or other forms of passive contamination were hypothesized as possible infection sources.
• Case highlights the importance of brucellosis screening in infants presenting with unexplained fever in endemic regions.

## 1 Introduction

Brucellosis, caused by the *Brucella* genus of bacteria, is an infectious zoonotic disease that poses serious global health threats, including in China [1]. *Brucella* spp., a group of tiny, gram-negative, facultative intracellular bacteria, exhibit high infectivity and a wide range of transmission routes and host spectrums [2]. The transmission routes of human brucellosis are diverse, including gastrointestinal exposure, mucosal contact, and respiratory inhalation, with direct contact with infected animal placenta or secretions constituting the primary transmission pattern in rural areas [3].

Human brucellosis is an infectious disease characterized by long-term fever, arthralgia, fatigue, acute sweating, enlargement of the liver and spleen, and local infections of various tissues and organs in the chronic course [4]. Although brucellosis mainly occurs in young and middle-aged agricultural populations, it frequently occurs through indirect or direct contact with animals and dairy products. However, owing to the geographic expansion of human brucellosis, its prevalence in non-occupational populations has notably increased in children [5]. In the case of family cluster brucellosis, children within a family can be infected in various ways, such as drinking raw goat milk and breastfeeding [6]. Moreover, cases of congenital brucellosis have been reported in Turkey [7], Saudi Arabia [8], and Kuwait [9], which are areas severely endemic for brucellosis [10]. The diagnosis of brucellosis is challenging in infants because it is characterized by nonspecific symptoms, lack of clinical suspicion, and rare exposure history, which can lead to misdiagnosis and delayed treatment [11], [12].

In most cases, congenital brucellosis is diagnosed by the unexpected isolation of *Brucella* from blood cultures obtained from a sick neonate with suspected sepsis [13]. Serologic tests are also important for clinical diagnosis; however, a negative serological test should not exclude the diagnosis, particularly in preterm neonates who may not have mounted their antibody response or received transplacental antibodies [14]. Therefore, timely and accurate diagnosis is key to managing brucellosis to obtain satisfactory diagnosis and treatment outcomes. Here, we describe a case of infantile brucellosis affecting the nervous system, aiming to improve the differential diagnosis of central nervous system infections in an endemic region.

## 2 Materials and methods

### 2.1 Epidemiological investigation

**Fig. 1:**
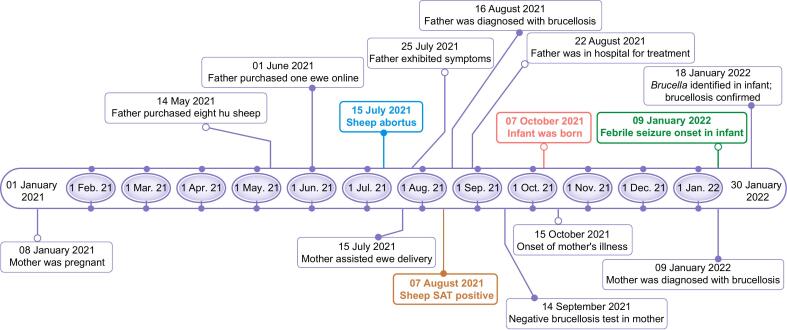
Flowchart of retrospective epidemiology survey of brucellosis transmission in a family cluster. Color-coded timeline illustrates key events: sheep abortus (blue), SAT-positive sheep (dark red), maternal childbirth (red), infant symptom onset (green). Abbreviation: SAT, standard tube agglutination test.

This case study involved a three-month-old infant residing in Xining City, Qinghai Province, China. On-site inquiries (patient demographics, clinical progression and therapeutic interventions, and symptomatology with physical signs), field visits (suspected exposure factors and the situation of their close contacts), and telephone surveys (the epidemiological connections among the three patients, infant’s recovery, and follow-up) were conducted to ascertain the source of infection. A case survey form was completed, and the survey content included basic patient information, exposure factors, and clinical manifestations. In addition, nine sheep and goats associated with the family were examined: eight Hu sheep (pregnant) were purchased from outside the province on May 14, 2021, and one female goat was purchased online in June (Fig. 1). Five Hu sheep were aborted in June 2021. Serological tests, including the Rose Bengal plate test (RBPT) and standard tube agglutination test (SAT), were performed to diagnose animal brucellosis. Local animal husbandry and veterinary department testing showed that eight (89 %) of nine sheep purchased from other provinces tested positive by SAT on August 7, 2021.

### 2.2 Case definition and laboratory testing

The case definition and laboratory examinations were performed according to the *Diagnostic Criteria for Human Brucellosis* (WS269-2019) in China [15]. Briefly, for a case to be confirmed, it must meet at least two of the following three criteria: 1) a history of epidemiological contact, 2) a positive reaction for RBPT, and/or 3) an SAT titer ≥1:100 (++), which is considered positive, or the *Brucella* strain must be isolated from patient samples. If the case meets the above diagnostic criteria but has no clinical manifestations, the case is considered a latent infection. RBPT, SAT, and bacterial culture were used to diagnose brucellosis. Briefly, blood samples were collected and centrifuged at 4,000 rpm for 10 min to separate the serum. RBPT and SAT were performed, and the results were interpreted according to standard approaches [16]. In the bacteriology approach, five samples (∼10 mL each) were collected from three suspected patients (mother, father, and infant), and cerebrospinal fluid (CSF) samples were obtained from the infant. The samples were injected into a culture bottle in a biosafety cabinet and incubated at 37 °C with 5 % CO_2_ for 2–4 weeks. The suspected strain was identified by matrix assisted laser desorption ionization-time of flight mass spectrometry (MALDI-TOF MS) (VITEK-MS system, France/BioMérieux) using the in-vitro diagnostic (IVD) knowledge database [17]. Briefly, colonies were picked, transferred directly to the target plate, ready-to-useα-cyano-4-hydroxycinnamic acid (CHCA) matrix, and loaded onto VITEK MS. All serology reagents were purchased from Qingdao Zhongchuang Biotechnology Co. Ltd. (China Manufacturer). In addition, routine blood tests, imaging examinations, and computed tomography (CT) were performed to assist in disease diagnosis.

## 3 Results

### 3.1 Epidemiology course and serological profiling

The infant’s father had raised sheep for over two years, with five Hu sheep experiencing abortions. In June 2021, the parents handled miscarriages without any protective measures. On July 25, the infant’s father experienced severe symptoms, including left-sided testicular pain, fever (peak temperature: 38.8 °C), sweating, and chills, prompting him to seek care at a local health clinic (Fig. 1). The infant’s father was subsequently diagnosed with brucellosis (RBPT-positive, SAT titer of 1:100) on August 16 (Fig. 1). The mother of the infant was pregnant on January 8, 2021 (Fig. 1). In mid-July, she assisted in delivering a ewe without protective measures. Serum tests later revealed that the ewe was infected with *Brucella* and was SAT-positive (Fig. 1). The mother experienced no clinical symptoms during pregnancy or childbirth, and many examinations during pregnancy were negative for brucellosis. However, she experienced excessive sweating and muscle and joint soreness on the eighth day after delivery (October 7) (Fig. 1). Her symptoms worsened on January 9, 2022, and her RBPT and SAT results were positive, with an SAT titer of 1:200 (++) (Fig. 1). The infant’s mother was admitted to the hospital for treatment, and the infant was weaned from the mother and fed milk powder. On January 9, 2022, the infant experienced febrile seizure that persisted for three days, reaching a maximum body temperature of 40.5 °C.

### 3.2 Microbiological and serological characterization

Routine blood tests revealed that the infant’s white blood cell count was 17.26 × 10^9^ cells/L, lymphocyte count was 55.7 %, neutrophil count was 30.2 %, red blood cell count was 4.6 × 10^12^ cells/L, hemoglobin level was 114 g/L, platelet count was 419 × 10^9^ cells/L, and C-reactive protein (CRP) level was 130.22 mg/L, all of which indicated the presence of infection. Head CT showed brain damage in the right frontal, parietal, and temporal lobes (polycystic encephalomalacia and atrophy) and bilateral apex frontal and left temporal subdural effusions. Chest CT (plain scan) indicated that the infant had pneumonia. Abdominal ultrasonography revealed no liver, gallbladder, spleen, pancreas, stomach, or bowel abnormalities. The procalcitonin and CRP levels in the CSF (105.46 mg/L) preliminarily suggested the presence of infection or inflammation.

### 3.3 Clinical management and therapeutic outcome

**Fig. 2:**
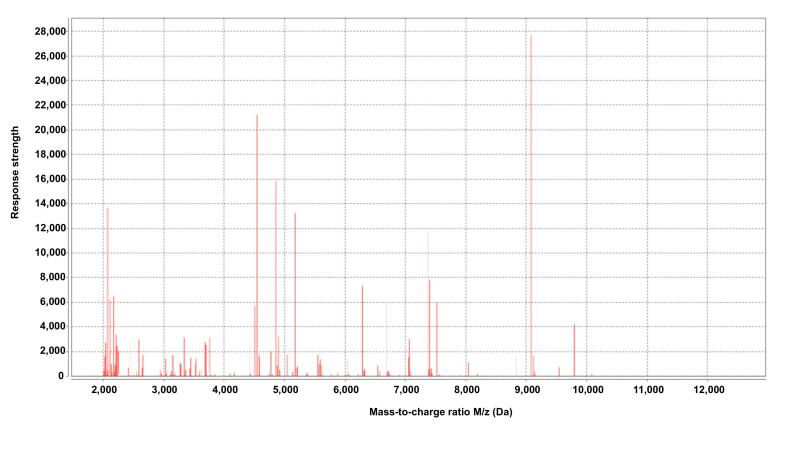
Mass spectrum profile of *Brucella* spp. in MALDI-TOF-MS (VITEK-MS) system. Abbreviation: MALDI-TOF-MS, matrix-assisted laser desorption/ionization time-of-flight mass spectrometry.

Following a comprehensive epidemiological investigation, both parents were confirmed as brucellosis cases (SAT positive) before the infant was diagnosed, and blood culture testing of the infant was performed immediately. After admission to the hospital, the infant was treated with a combination of cefoperazone sodium, amoxicillin, and sulbactam to treat a fever of unknown origin. The CSF culture was negative, and *Brucella* strains were isolated from a blood sample on January 18, 2022, and identified as *Brucella* spp. by MALDI-TOF-MS (Fig. 2) with a confidence score of 99.9 %; the species/biovar of the strain was not determined. Infantile meningitis caused by *Brucella* strain was confirmed. During follow-up after treatment, brucellosis examination showed that the RBPT was positive and the SAT titer was 1:200 (+ +). The infant had engaged in no outdoor activities within the past three months, and the parents denied any contact with cattle or sheep; breastfeeding was the only source of diet. After confirming the *Brucella* strain infection, antibiotics were adjusted immediately and treated with ceftriaxone sodium (2.0 g/day) and rifampicin (0.5 g/day) for 4 weeks. The biochemical indices of the CSF examination returned to normal (6.8 mg/L), fever (36.9 °C) or convulsions disappeared, the illness greatly improved, and there were no significant complications or signs of relapse within two years after the initial treatment; subsequently, the status of the patient has not been traced.

## 4 Discussion

Although the incidence of brucellosis in the nervous system is low (0.5 %–25.0 %) [18], the central and peripheral nervous systems can be involved, and meningitis may be present [19]. In this study, a three-month-old infant without any history of high-risk exposure to *Brucella* or ingestion of raw milk and whose only source of nutrition was breastfeeding developed brucellosis. The infant’s father had been diagnosed with brucellosis three months prior, but the infant’s mother had not contracted brucellosis during pregnancy. Although transmission to an infant typically occurs through the placenta or breastfeeding, it is highly likely that the infection can also be acquired from the parent’s hands, clothes, or other sources of passive contamination. A previous report of congenital brucellosis described late prenatal transmission from a mother to her term infant [20]. In another report described a case of *B. melitensis* infection in an obstetrician who was infected during the delivery of an infant with congenital brucellosis [21]. It has been found that direct contact with soil, animal feces, and dust contaminated with *Brucella* is associated with a higher risk of infection. Vertical transmission, sexual transmission, and breast milk transmission are rarely reported routes of infection [22]. Thus, the infant becomes infected by inhaling infected aerosolized particles or by direct transmission via mucosal surfaces [23]. This case implies that *Brucella* may even affect people not directly exposed to infected animals through contaminated material and the environment. In addition, brucellosis is concentrated in adult breeding animals domestically and in contact with animals; therefore, pediatricians may not be aware of the clinical manifestations of childhood brucellosis [24]. Due to the variable clinical features of the disease, unfamiliarity with brucellosis can delay diagnosis in children who are not occupationally exposed [25].

In this study, the *Brucella* strain was isolated from the blood sample of an infant, but the CSF culture was negative. Neurobrucellosis (NB) is often difficult to diagnose because the titer of specific anti-*Brucella* antibodies in CSF is usually low, and the yield of the cultures is poor, with positivity rates of only 40 %–50 % [26]. CSF cultures of *Brucella* were performed in eight patients, of which two were positive (25.00 %) and six were negative (75.00 %) [27]. This evidence indicates that isolation of *Brucella* remains a valuable method for diagnosing infant brucellosis at an early stage. In addition, PCR has been used in cases where diagnosis of NB has been challenging and in cases of seronegative organ-specific brucellosis. PCR assays in CSF samples are more rapid and sensitive than conventional microbiological tests [28].

This study is the initial CSF examination that revealed elevated protein levels, normal or low glucose levels, and lymphocytic leukocytosis. In one study, high levels of CRP, aspartate aminotransferase, and alanine aminotransferase were significant in the bacteremia group [29]. In another study of 43 patients diagnosed with brucellosis in Iran, anemia (65 %), lymphocytosis (51 %), elevated erythrocyte sedimentation rate (86 %), and elevated CRP level (67 %) were the most prominent blood anomalies [30]. These data indicate high protein and low glucose concentrations in the CSF are potential biomarkers for infant brucellosis meningitis.

This study used ceftriaxone sodium and rifampicin to treat infant brucellosis meningitis, and recovery was obtained after four continuous weeks of medication administration. To treat brucellosis meningitis, a combination of three antibiotics and drugs that smoothly pass through the blood–brain barrier, such as rifampicin, ceftriaxone sodium, and cotrimoxazole, should be selected [31]. Norfloxacin, tetracycline, and other antibiotics that affect pediatric bone development and risk staining are contraindicated in children [32], [33]. Brucellosis has variable presentation, lacks specificity, has clinical symptoms similar to a variety of diseases, which makes clinical diagnosis and treatment difficult [34], [35]. Although NB is rare in infants, it is considered in the differential diagnosis of central nervous system infection, in which a simplified brucellosis serum screen is a priority, especially in endemic regions.

## 5 Conclusion

In this survey, the detailed transmission route of infant infection by *Brucella* strain could not be determined, maternal-fetal transmission or contamination through breastfeeding, parental hand contact, clothing exposure, or other passive contamination modes may be potential transmission routes. Such a possibility strengthens the need to increase the awareness and education of parents in endemic areas so that they can gain an understanding of the risk factors of disease in children in families with domestic animals and encourage them to avoid unnecessary direct contact with cattle, sheep, and other livestock and their excrement. Primary medical staff should be trained to prevent and treat brucellosis to avoid misdiagnoses.

## Ethics statement

This study was conducted under the principles of the Declaration of Helsinki and approved by the Ethics Committee of Qinghai Provincial Women and Children’s Hospital (2022-wjzdx-38), which was a retrospective study of a case of brucellosis meningitis in a three-month-old infant in Xining City, Qinghai Province. Informed consent was obtained from the patient’s parents, and *Brucella* spp. was isolated. Strains were used to diagnose brucellosis. The owners of the animals approved and consented verbally to their participation in the study.

## Acknowledgements

This study was supported by the Guiding Project of Qinghai Health System (2022-wjzdx-38). The funders played no role in the study design, data collection and analysis, publication decision, or manuscript preparation.

## Conflict of interest statement

The authors declare that there are no conflicts of interest.

## Author contributions

**Guowu Shen:** Writing – original draft, Resources, Methodology, Investigation, Formal analysis. **Xiaohua Zhao:** Writing – review & editing, Methodology, Investigation, Data curation. **Jie Chen:** Writing – original draft, Visualization. **Xuehui Zhang:** Writing – review & editing, Supervision, Methodology, Formal analysis, Data curation. **Xin Wang:** Writing – review & editing, Resources, Methodology, Investigation, Data curation. **Zhiguo Liu:** Writing – review & editing, Writing – original draft, Visualization, Methodology, Investigation, Formal analysis, Supervision, Conceptualization. **Zhenjun Li:** Writing – review & editing, Writing – original draft, Validation, Supervision, Methodology, Funding acquisition, Conceptualization. **Canjun Zheng:** Writing – review & editing, Validation, Investigation, Conceptualization.
